# Antioxidant properties, ACE/renin inhibitory activities of pigeon pea hydrolysates and effects on systolic blood pressure of spontaneously hypertensive rats

**DOI:** 10.1002/fsn3.740

**Published:** 2018-08-22

**Authors:** Aderonke I. Olagunju, Olufunmilayo S. Omoba, Victor N. Enujiugha, Adeola M. Alashi, Rotimi E. Aluko

**Affiliations:** ^1^ Department of Food Science and Technology Federal University of Technology Akure Ondo State Nigeria; ^2^ Department of Food and Human Nutritional Sciences University of Manitoba Winnipeg MB Canada

**Keywords:** antihypertensive activity, free radical scavenging, pancreatin, pepsin, pigeon pea hydrolysates

## Abstract

Legumes are rich sources of protein in human diet and their consumption has been associated with the prevention of chronic diseases attributable to their bioactive components. Pigeon pea (*Cajanus cajan*) is an underutilized legume with relatively high protein content (~24%). Protein hydrolysates were prepared from pea isolate by enzymatic hydrolysis using pepsin and pancreatin. Hydrolysates were evaluated for their amino acid composition, antioxidant properties, in vitro and in vivo antihypertensive properties. The hydrolysates had high hydrophobic amino acids, especially isoleucine, phenylalanine, and leucine. Pepsin‐pancreatin‐hydrolyzed pea protein (PPHPp) showed significantly higher ability to scavenge DPPH˙ while pancreatin‐hydrolyzed pea protein (PPHPa) had higher ˙OH, ABTS˙^+^ scavenging, Fe^3+^ reducing and linoleic acid peroxidation inhibition. PPHPp exhibited superior angiotensin‐converting enzyme inhibition (61.82%) while PPHPa showed higher renin inhibition (14.28%). PPHPp exhibited strong antihypertensive effect, showing an instantaneous systolic blood pressure lowering effect (−26.12 mmHg) within 2‐h post‐oral administration. Pigeon pea protein hydrolysate (especially from pancreatin digest) could therefore, be a promising source of bioactive peptides and potential ingredient for formulation of functional foods against oxidative stress and hypertension.

**Research highlights**

- Bioactive peptides were obtained from pigeon pea via enzymatic hydrolysis
- Pigeon pea peptides showed good inhibitory activity against ACE and renin
- Hydrolysates exhibited systolic blood pressure reduction in hypertensive rats

## 1 INTRODUCTION

Reactive oxygen species (ROS) are produced during normal metabolism and from exogenous sources such as air pollutants, ionizing radiation, and carcinogenic compounds (Birben, Sahiner, Sackesen, Erzurum, & Kalayci, 2012; Halliwell, 2007). ROS can initiate the oxidation of biomolecules, leading to tissue damage, mutagenic changes, and eventually cell death. Oxidative stress, resulting from an imbalance between ROS and antioxidant defense, plays a pivotal role in the development of degenerative diseases such as cancer and hypertension (Ceriello, 2008). Antioxidants prevent oxidative tissue damage by quenching free radicals and chelating prooxidant metal ions. Antioxidant peptides remain encrypted in the amino acid sequence of parent protein as inactive fragments. They are usually released via enzyme‐catalyzed hydrolysis, fermentation and or gastrointestinal digestion after oral ingestion by human. Food‐derived antioxidants from hydrolysates have been of major research interest being rich sources of peptides with physiological antioxidative effects and prevention of oxidative food degradation. They serve as alternatives to existing synthetic antioxidants (butylated hydroxylanisole, butylated hydroxytoluene) whose usage is associated with undesirable and adverse side effects.

The physiological blood pressure is regulated by the renin‐angiotensin system (RAS). Renin (produced in the kidney) converts angiotensinogen (in the liver) to a decapeptide, angiotensin I, which is biologically inactive, but activated by conversion to angiotensin II (a major vasoconstrictor) by angiotensin‐converting enzyme (ACE). Persistent high blood pressure results from uncontrolled release of angiotensin II, and is a controllable risk factor for cardiovascular diseases such as stroke and heart failure, which are the leading causes of death and disability worldwide (Jensen, Eysturskarö, Madetoja, & Eilertsen, 2014). Inhibition of enzymes in the RAS pathway, especially ACE, is regarded to be a potent therapeutic approach in the treatment of hypertension (Lin, Lv, & Li, 2012). ACE inhibition functions to block the first step in the renin‐angiotensin system thereby interrupting the negative feedback effects of angiotensin II. Synthetic ACE inhibitors (captopril, lisinopril, enalapril) extensively used as antihypertensive drugs are available on the market, but their use is associated with adverse effects and nontolerance in some patients. ACE inhibitory peptides can be derived from different plant sources and may function as potent alternatives to synthetic drugs as they have been adjudged to be safe and economical. Legumes have prospects for the release of bioactive peptides with diverse physiological activities (Torruco‐Uco, Chel, Martínez, Dávila, & Betancur, 2009). Commonly cultivated legumes have been exploited in this regard, leaving the underutilized legumes underexploited with limited information on their nutraceutical potential in management of key chronic disease and as functional ingredients in nutraceutical industry.

Based on the demonstrated relationship between oxidative stress and high blood pressure, the use of a single agent with antioxidant and antihypertensive properties could provide an effective strategy for the prevention and treatment of hypertension. Pigeon pea is an underutilized legume in Nigeria, used as food and forage, a good source of protein (20–28%). It used to be a delight and delicacy about two decades ago but presently tending toward extinction owing to scanty information on its nutritional and nutraceutical potential compared to other common legumes (bambara, cowpea, African yam bean, etc.).

Therefore, the objective of this study was to evaluate pigeon pea protein hydrolysates for antioxidative capacity, as well as in vitro and in vivo antihypertensive properties.

## 2 MATERIALS AND METHODS

### 2.1 Materials

Pigeon pea (*Cajanus cajan*) seed (TCc‐AO/TB78‐9) was obtained from the Gene bank of the International Institute of Tropical Agriculture, Ibadan, Oyo State, Nigeria. Reagents used were purchased from Sigma (St. Louis, MO, USA) and Fisher Scientific (Oakville, ON, Canada).

### 2.2 Preparation of pigeon pea protein isolate (PPI)

Pigeon pea isolate was obtained by isoelectric precipitation. Pigeon pea flour (90 g) was suspended in 1800 ml of double distilled water (DDQ), adjusted to pH 9.0 using 1 M NaOH and extracted for 1 h at 25°C on a stirrer plate. pH was adjusted to 4.5 using 1 M HCl solution. The precipitate was centrifuged (9000 *g*; 30 min; 4°C), washed with DDQ thrice, adjusted to pH 7.0, lyophilized and stored at −20°C.

### 2.3 Enzymatic hydrolysis of PPI

PPI (5%, w/v) was hydrolyzed with pancreatin (pH 8.0, 37°C) and pepsin (pH 2.0, 37°C) for 4 h at enzyme to substrate ratio of 1:20 (w/w). Also, a sequential hydrolysis with pepsin followed by pancreatin for 2 h each was done. The pH was maintained during each hydrolysis using either 1 M NaOH or 1 M HCl as appropriate, while the temperature was maintained using a thermostat. The enzymes were inactivated after hydrolysis, by heating and holding at 85°C for 15 min. The cooled reaction mixtures were centrifuged (9000 *g*; 30 min; 4°C), supernatant lyophilized and stored at −20°C.

### 2.4 Determination of amino acid composition

The amino acid profiles were determined using the HPLC Pico‐Tag system according to the method previously described after samples were digested with 6 M HCl for 24 h (Bidlingmeyer, Cohen, & Tarvin, 1984). The cysteine and methionine contents were determined after performic acid oxidation and the tryptophan content was determined after alkaline hydrolysis.

### 2.5 Determination of antioxidant properties

DPPH radical scavenging activity was determined by the method of by Girgih, Udenigwe, Li, Adebiyi, and Aluko (2011) with slight modifications for a 96‐well flat bottom plate. Samples were dissolved in 0.1 M sodium phosphate buffer, pH 7.0 containing 1% (v/v) Triton‐X. DPPH was dissolved in methanol to a final concentration of 100 μM. One hundred microlitre (100 μl) aliquot of each sample was mixed with 100 μl of the DPPH radical solution in a 96‐well plate to final concentrations of 0.5–4 mg/ml and incubated at room temperature in the dark for 30 min. The buffer was used in the blank assay while reduced glutathione (GSH) served as the positive control. Absorbance was measured at 517 nm using a spectrophotometer and the percentage DPPH radical scavenging activity was determined using the following equation:

$$\text{DRSA}=\frac{\text{Absorbance (blank)}-\text{Absorbance (sample)}}{\text{Absorbance (blank)}}\times 100$$

Hydroxyl radical scavenging activity (HRSA) was also based on a method described by Girgih et al. (2011) with modifications. Samples, GSH and 1, 10‐phenanthroline (3 mM) were each separately dissolved in 0.1 M phosphate buffer (pH 7.4) while FeSO_4_ (3 mM) and 0.01% hydrogen peroxide were each separately dissolved in distilled water. An aliquot (50 μl) of sample or GSH (equivalent to a final assay concentration of 1 mg/ml) or buffer (control) was first added to a clear, flat bottom 96‐well plate followed by additions of 50 μl of 1, 10‐phenanthroline and 50 μl of FeSO_4_. To initiate reaction in the wells, 50 μl of hydrogen peroxide (H_2_O_2_) solution was added to the mixture, which was then covered and incubated at 37°C for 1 h with shaking. Thereafter, the absorbance of the mixtures was measured at 536 nm every 10 min for a period of 1 h. The hydroxyl radical scavenging activity was calculated as follows based on change in absorbance (Δ*A*):

$$\text{HRSA}=\frac{\Delta A/\min\;(\text{control})-\Delta A/\min\;(\text{sample})}{\Delta A/\min\;(\text{control})}\times 100$$

The ferric reducing antioxidant power (FRAP) of the protein was determined according to the modified method of Benzie and Strain (1996). Peptide or GSH was dissolved in 0.3 M acetate buffer (pH 6.6). The FRAP reagent was prepared by mixing 0.3 M acetate buffer with 10 mM TPTZ (2,4,6 tripyridyl‐s‐triazine) at pH 3.6 in 40 mM HCl and 20 mM FeCl_3_.6H_2_O pH 3.6, at the ratio of 5:1:1 (v/v/v), respectively. The isolate and hydrolysates were dissolved in distilled water. Solutions of FeSO_4_·7H_2_O in the range of concentrations of 0.0625–1.0 μmol/μl were used for the calibration. The samples (40 μl) were placed into a clear 96‐well plate and 200 μl of the FRAP reagent added. The reaction was carried out at 37°C and the absorbance was read at 593 nm. The results were expressed in mmol of Fe^2+^ per mg of sample based on the calibration curve.

ABTS radical scavenging activity (ARSA) was carried out according to a previously described method (Arts, Dallinga, Voss, Haenen, & Bast, 2004). Briefly, ABTS^+^ was prepared by dissolving 7 mM ABTS and 2.45 mM potassium persulphate in phosphate buffered saline (PBS), pH 7.4 and allowing this to stand in the dark for 16 h to generate the ABTS radical cation (ABTS˙^+^). For the analysis, the ABTS˙^+^ stock was diluted using PBS buffer and equilibrated at 30°C to an absorbance of 0.7 (±0.02) at 734 nm using a Heliosk thermo spectrophotometer (Electron Corporation Helios Gamma, England). Trolox (6‐hydroxy‐2,5,7,8‐tetramethylchroman‐2‐carboxylic acid) was dissolved in 80% ethanol. The antioxidant capacity was measured by mixing 200 μl of samples with 2 ml of ABTS^+^ solution and the decline in absorbance was observed for 5 min. Appropriate blanks were run for each sample and the radical scavenging capacity was compared to that of Trolox (6.25–200 μM) and results were expressed as mM Trolox equivalent (TE) per gram of sample on protein equivalent basis. The percentage ABTS˙^+^ scavenged was calculated using the following equation:

$$\begin{array}{cc} \text{ABTS radical scavenging activity} & =\frac{\Delta A/\min\;(\text{control})-\Delta A/\min\;(\text{sample})}{\Delta A/\min\;(\text{control})}\\ & \;\;\;\;\;\;\;\times 100 \end{array}$$

### 2.6 Determination of linoleic acid oxidation

Linoleic acid oxidation was determined using the method described by Girgih et al. (2011). Samples or GSH were each dissolved in 1.5 ml of 0.1 M phosphate buffer, pH 7.0. Each sample solution was added (final assay concentration of 0.5 mg/ml) to 1 ml of 50 mM ethanolic linoleic acid and stored in a glass test tube kept at 60°C in the dark for 7 days. On a daily basis, 100 μl of the sample mixture was removed and mixed with 4.7 ml of 75% aqueous ethanol, 0.1 ml of ammonium thiocyanate (30%, w/v) and 0.1 ml of 0.02 M acidified ferrous chloride (dissolved in 1 M HCl). An aliquot (200 μl) of the resulting mixture was added to a clear bottom 96‐well plate and the degree of color development was measured at 500 nm after 3 min incubation at room temperature.

### 2.7 Determination of angiotensin I‐converting enzyme (ACE) inhibitory activity

The inhibition of ACE was determined as described by Udenigwe, Lin, Hou, and Aluko (2009), using N‐(3‐[2‐furyl] acryloyl)‐phenylalanylglycylglycine (FAPGG), as substrate. An aliquot (1 ml) of FAPGG (0.5 mM, dissolved in 50 mM Tris‐HCl buffer containing 0.3 mM NaCl, pH 7.5) was mixed with 20 μl of 1 U/ml ACE (final assay activity was 20 mU) and 200 μl of sample solution (prepared in the Tris‐HCl buffer). Cleavage of the Phe‐Gly peptide bond in FAPGG results in a decrease in absorbance at 345 nm, which was recorded every 10 s for 2 min at room temperature. For control (uninhibited) reaction, peptide sample was omitted from the reaction mixture.

ACE inhibition was calculated as follows:

$$\%\;\text{ACE inhibition}=\frac{\text{Slope (Blank)}-\text{Slope (sample)}}{\text{Slope (blank)}}\times 100$$

### 2.8 Determination of renin inhibitory activity

The ability of peptide samples to inhibit renin activity was determined according to a previously described method (Li & Aluko, 2010) using the Renin Inhibitor Screening Assay Kit. The assay mixture (190 μl) consisted of 10 μM Arg‐Glu (EDANS) ‐Ile‐His‐Pro‐Phe‐His‐Leu‐Val‐Ile‐His‐Leu‐Val‐ile‐His‐Thr‐Lys (Dabcyl)‐Arg (renin substrate in dimethyl sulfoxide), human recombinant renin and peptides, all dissolved in 50 Mm Tris–HCl buffer, pH 8.0 containing 100 mM NaCl. For the control experiment, peptide sample was omitted from the reaction mixture. The renin substrate was mixed with the peptide sample and incubated at 37°C for 10 min to attain thermal equilibrium. Enzyme reaction was initiated by addition of human recombinant renin to the mixture, followed by recording of the increase in fluorescence intensity for 10 min in a thermostated Spectra Max Gemini Fluoresence Microplate Reader spectrofluorometer (Molecular Devices Sunnyvale, CA). The spectrofluorometer was set at excitation and emission wavelengths of 340 and 490 nm, respectively. The enzyme activity was expressed as reaction rate, arbitrary fluorescence intensity unit per minute (FIU min^−1^).

$$\text{Renin inhibition}=\frac{\text{FI}\;(\text{Blank})-\text{FI}\;(\text{sample well})}{\text{FI (control well)}}\times 100$$

### 2.9 Antihypertensive activity study using spontaneously hypertensive rats (SHRs)

Animal experiments were performed according to protocols approved by the University of Manitoba Animal Ethics Committee with protocol number F16‐008/1. Male SHRs were purchased at 6 weeks from Charles River (Montreal, PQ, Canada) allowed one week of acclimatization, implanted with Data Sciences International (DSI) HD‐S10 telemetry transmitters (DSI, St. Paul, MN, USA). Rats were orally gavaged with 1 ml of solution using a disposable plastic syringe. Real‐time systolic blood pressure (SBP) measurements was collected in a quiet room with each rat cage placed on top of the receiver (Model RPC‐1, DSI instruments, MN, USA) assigned to the implant. Data were recorded continuously at 10 min intervals for 24 h using Ponemah 6.1 data acquisition software (DSI instruments, MN, USA). An APR‐1 atmospheric‐pressure monitor (DSI instruments, MN, USA) was attached to the system to normalize the transmitted pressure values so that the recorded blood pressure signals are independent of atmospheric pressure changes.

### 2.10 Statistical analysis

Data were generated in triplicates and subjected to Analysis of Variance (ANOVA) using Statistical Package for Social Sciences (SPSS) V. 17.0. The means were separated using Duncan Multiple Range Test (DMRT) at 95% confidence level.

## 3 RESULTS AND DISCUSSION

### 3.1 Amino acid composition of pigeon pea protein and hydrolysates

The amino acid composition of pigeon pea isolate and hydrolysate showed that glutamic acid, aspartic acid, lysine, leucine, arginine, and phenylalanine were predominant (Table 1). Amino acid profile of pepsin‐pancreatin hydrolyzed protein (PPHPp) compared closely with those reported by other researchers for *Phaseolus lunatus* hydrolyzed using same enzyme (Magaña, Segura‐Campos, Dávila‐Ortiz, Betancur‐Ancona, & Chel‐Guerrero, 2015). The histidine, lysine, cysteine, and phenylalanine content of PPHPa and pepsin‐hydrolyzed protein (PPHPe) are significantly higher than the amount observed in canola hydrolysate obtained using pancreatin and pepsin enzymes, respectively (Alashi et al., 2014). The content of leucine and isoleucine residues as well as hydrophobic aromatic amino acids (phenylalanine and tyrosine) were highest in PPHPe and PPHPp. Hydrolysis with different proteases did not have significant effect on the amino acid composition of the hydrolysates. Although, some specific amino acids such as valine, glutamic acid, and tryptophan were higher in the hydrolysates compared to the intact protein isolate. The difference in the amino acid composition in the different hydrolysates may be associated with the unique specificity of the various protease employed. PPHPe and PPHPp showed high content of hydrophobic amino acid (HAA) and aromatic amino acid (AAA), respectively. The HAA observed was higher than 22.33 g/100 g reported for cowpea isolate. HAA have been reported to have a relationship with antioxidant activity (Valdez‐Ortiz, Fuentes‐Gutiérrez, Germán‐Báez, Gutiérrez‐Dorado, & Medina‐Godoy, 2012). Hydrophobicity of peptides increase peptide solubility and facilitates interaction and proton exchange with radical species (Zou, He, Li, Tang, & Xia, 2016). The pea proteins obtained in this study exhibited considerable content of the hydrophobic amino acids especially isoleucine (3.46–3.68 g/100 g), leucine (6.88–8.37 g/100 g), phenylalanine (6.80–8.58 g/100 g), glycine (3.12–3.63 g/100 g), alanine (3.82–4.04 g/100 g), and proline (4.83–5.06 g/100 g). AAAs are known to freely donate hydrogen atom to electron deficient free radicals hence neutralizing the radical as well as breaking the radical chain. The presence of AAA has also been reported to enhance antioxidant capacity of peptides (Girgih et al., 2014). Cysteine with sulfhydryl (SH) group can also donate hydrogen atom from the SH group. Hence, the presence of cysteine and tryptophan in the same peptide chain would make a significant contribution to the antioxidant activities of such peptide (Zou et al., 2016).

**Table 1:** Amino acid composition of pigeon pea isolate and hydrolysates

Composition (g/100 g)					
AA/samples	PPI	PPHPe	PPHPa	PPHPp	FAO/WHO (2007)
Aspartic acid	11.55	11.59	9.53	10.26	
Glutamic acid	20.06	19.90	22.50	21.41	
Serine	6.19	6.27	5.60	5.79	
Proline	4.83	5.02	5.04	5.06	
Glycine	3.12	3.63	3.54	3.61	
Alanine	4.00	4.04	3.82	3.86	
Arginine	6.91	6.95	7.57	7.36	
Valine	3.46	3.87	3.76	3.81	3.90
Methionine	0.97	0.90	0.75	0.82	1.60
Cysteine	0.43	0.60	0.59	0.60	0.60
Isoleucine	3.68	3.48	3.46	3.52	3.00
Leucine	8.37	8.09	6.88	7.34	5.90
Tyrosine	2.73	2.77	2.60	2.74	
Phenylalanine	8.58	7.38	6.80	7.37	3.80
Histidine	4.61	3.98	6.24	5.31	1.50
Threonine	3.27	3.93	3.43	3.66	2.30
Lysine	7.05	7.36	7.56	7.18	4.50
Tryptophan	0.20	0.25	0.32	0.30	0.60
HAA	37.24	36.38	34.02	35.42	
AAA	11.51	10.39	9.72	10.40	

*Note*.

HAA: Hydrophobic Amino Acids; AAA: Aromatic Amino Acids; FAO: Food and Agriculture Organization; WHO: World Health Organization.

The presence of peptides with significant aromatic residues (tyrosine, phenylalanine, tryptophan) at C‐terminal and basic residues (lysine, histidine, arginine) at the N‐terminal have been suggested to have strong and competitive ACE inhibitory activity (Hwang & Ko, 2004). AAA also contribute as inhibitors of Angiotensin Converting Enzyme (ACE) by interacting between three subsites at the active site of ACE.

The result of the present study therefore, suggests that the presence of aromatic and hydrophobic amino acids in pigeon pea proteins is a valuable contributor to its antioxidative and antihypertensive properties.

### 3.2 Antioxidant properties of pigeon pea protein hydrolysate

**Figure 1:**
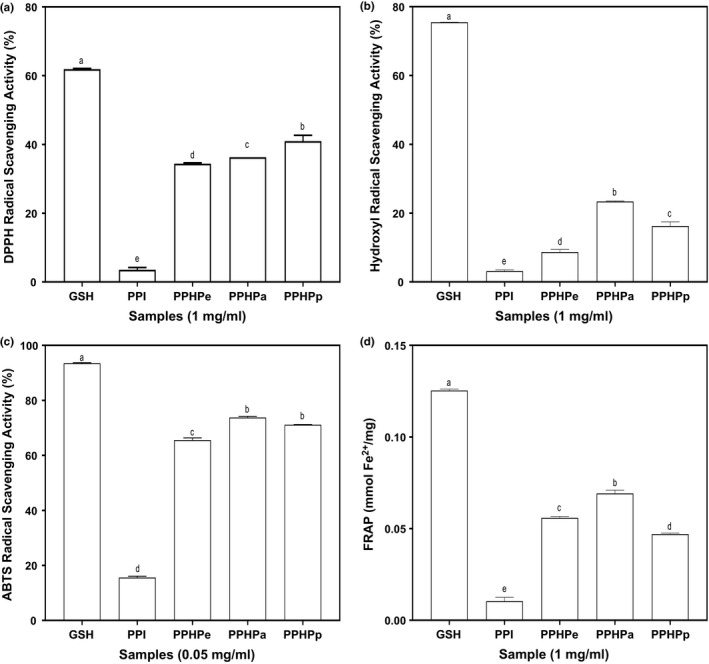
Radical scavenging activity of pigeon pea hydrolysates. Values are mean ± SD; Bars with different letters are significantly different at *p* < 0.05. (a) DPPH radical scavenging activity; (b) Hydroxyl radical scavenging activity; (c) ABTS radical scavenging activity; (d) Ferric Reducing Antioxidant Power. GSH: Glutathione; PPI: Pigeon pea protein isolate; PPHPe: Pepsin hydrolyzed pigeon pea protein; PPHPa: Pancreatin hydrolyzed pigeon pea protein; PPHPp: Pepsi‐pancreatin hydrolyzed pigeon pea protein

DPPH˙ is used to measure the ability of antioxidative compounds to donate electrons or hydrogen ion to free radicals to form a more stable compound. DPPH˙ receives hydrogen from antioxidant to form a stable diamagnetic molecule (yellow‐colored diphenyl picrylhydrazyl), and the extent of discoloration of the radical from purple to yellow indicates the scavenging potential of the antioxidant compound with respect to its hydrogen‐donating ability. The protein hydrolysates had better DPPH˙ scavenging activity than the intact protein (PPI), and thus indicates the release and exposure of peptides with enhanced radical scavenging activity from the isolate during enzymatic proteolysis (Figure 1a). However, activities of the protein isolate and hydrolysates (3.54–40.98%) were significantly lower than that of GSH an excellent free radical scavenger (Galano & Alvarez‐Idaboy, 2011). PPHPp had significantly (*p* < 0.05) higher DPPH˙ scavenging ability than the other hydrolysates. A previous work has also shown that a rapeseed hydrolysate produced with pepsin‐pancreatin had the highest DPPH˙ scavenging activity when compared to hydrolysates from other enzymes (He, Girgih, Malomo, Ju, & Aluko, 2013). Moreover, extensive hydrolysis with pepsin‐pancreatin would likely release smaller sized peptides that can easily access their targets in the reaction matrix. The results obtained in this study for PPHPa (36.30%) are lower than the 61% reported for pancreatin‐hydrolyzed Azufrado protein (Valdez‐Ortiz et al., 2012). The DRSA for PPHPe (34.42%) is however, higher than the 4% reported for hemp seed hydrolysate obtained using pepsin (Girgih, Udenigwe, Li, & Aluko, 2013).

Hydroxyl radical can be generated from superoxide radical and it is considered the most damaging specie in oxidative stress due to its high oxidizing power. The hydroxyl radical can cause several damages by reacting with target molecules, which are vital for normal cell functioning. At 1 mg/ml, the hydroxyl radical scavenging activity (HRSA) values are 3.13, 8.68, 23.36, and 16.23% for PPI, PPHPe, PPHPa, and PPHPp, respectively (Figure 1b). The fact that enzymatic hydrolysis using the various proteases employed significantly increased the hydroxyl radical scavenging potential of pigeon pea protein can be deduced from the result. However, PPHPa (23.36%) significantly (*p* < 0.05) exhibited superior HRSA than the other hydrolysates (PPHPe and PPHPp). The HRSA obtained for pancreatin hydrolysate (PPHPa) in this study was similar to 22.50% reported by Ralison, Tounkara, Karangwa, Yong, and Le (2013) for black soybean hydrolyzed with pancreatin. Girgih et al. (2013) on the other hand reported no activity for pepsin‐hydrolyzed hemp seed protein. Ajibola, Fashakin, Fagbemi, and Aluko (2011) in their reports associated the HRSA and antioxidant activity of a peptide to its hydrophobicity. However, results from this present study showed an independent relationship between hydrophobic character of the pigeon pea hydrolysates and ability to scavenge hydroxyl radical.

Results of ABTS˙^+^ scavenging activity is presented in Figure 1c. There was no significant difference (*p* < 0.05) between scavenging activity of PPHPa (73.8%) and PPHPp (71.1%). All the hydrolysates evaluated showed high ABTS˙^+^ antiradical activity. Similar to other results on radical scavenging activity of pigeon pea protein, protein isolate exhibited the lowest ABTS˙^+^ scavenging activity. Relative to the amino acid composition of the isolate and hydrolysates, PPI had the highest hydrophobic amino acid. High amounts of hydrophobic amino acids have been associated with low solubility which induces low ABTS radical scavenging activity (Zou et al., 2016). ABTS method showed to be a more sensitive method of determining antioxidant capacity of protein samples as the EC_50_ (μg/ml) obtained (data not shown) were lower than results of DPPH method (mg/ml). This suggests that ABTS method can determine antioxidant capacity at lower inhibition concentration. Valdez‐Ortiz et al. (2012) reported 73 and 75.3% for ABTS˙^+^ scavenging activity of Azufrado protein hydrolysate obtained using pepsin and pancreatin, respectively. The results of this study corroborate the reports of Alashi et al. (2014) where ABTS˙^+^ scavenging activity of canola protein hydrolysate using pancreatin enzyme was more effective than those of pepsin.

Antioxidants donate hydrogen atom to electron deficient free radical and this electron donating ability can be evaluated using ferric reducing antioxidant power. The antioxidant activity of a peptide directly correlates to its ferric ion reducing ability. The ability of pigeon pea hydrolysates to reduce Fe^3+^ ranged from 0.027 to 0.069 mmol Fe^2+^/mg, and was comparable to that of GSH (Figure 1d). Enzymatic hydrolysis significantly increased the reducing power of the pea protein. It was also observed that irrespective of the protease employed for enzymatic hydrolysis and their individual specificities, the hydrolysates acted as stronger reducing agent than the crude isolate. The ability of the peptide to reduce Fe^3+^ was highest in PPHPa (0.07 mmol Fe^2+^/mg). Fe^3+^ reduction is often used as an indicator of electron donating ability, which is an important mechanism of phenolic antioxidant action. He et al. (2013) reported no detectable FRAP for rapeseed hydrolysates. Similar to the result of the present study, Karamać, Kosinska‐Cagnazzo, and Kulczyk (2016) reported highest Fe^3+^ reducing ability for flaxseed hydrolysate using pancreatin. Antioxidants exert their reducing ability by breaking the free radical chain via donating a hydrogen atom. The results indicate that enzyme hydrolysis of pigeon pea protein led to the release of peptides with greater FRAP than the native protein molecules.

There was no correlation between ferric reducing antioxidant power and ABTS˙^+^ scavenging activity as the type of radical and their mechanism of reaction differs. The ferric reducing antioxidant power shows the reducing potential of an antioxidant (peptide) when it reacts with Fe^3+^‐TPTZ complex resulting in a deep blue colored compound (Fe^2+^‐TPTZ). In this reaction, the peptide serves as a hydrogen atom donator to stabilize or neutralize the free radical. Moreso, the reaction is conducted under acidic condition of pH 3.6 so as to iron solubility. On the other hand, ABTS˙^+^ scavenging activity was conducted at pH 7.4 and ABTS˙^+^ serves as the oxidant and produces a green colored compound in which the extent of decolorization of the color relates with the percentage inhibition of radical or its scavenging ability.

### 3.3 Inhibition of linoleic acid oxidation

**Figure 2:**
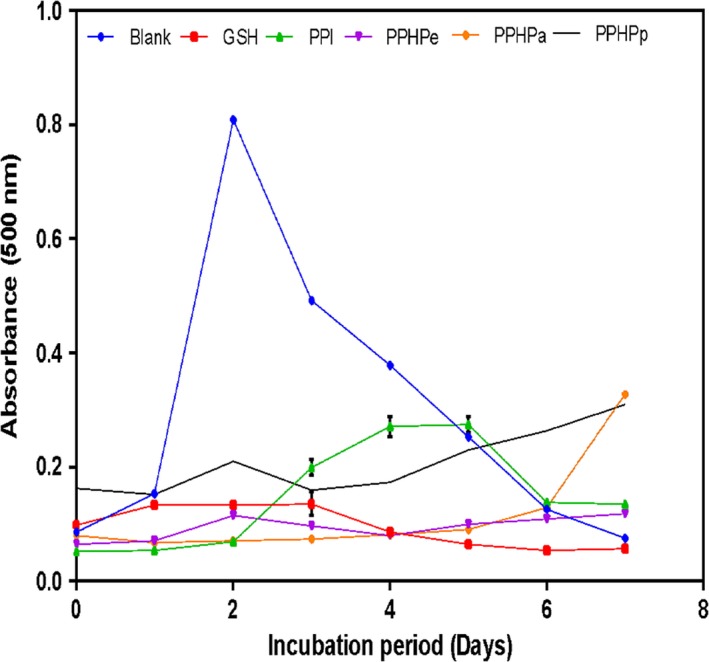
Inhibition of linoleic acid oxidation of pigeon pea protein isolate and hydrolysates. Values are mean ± SD. GSH: Glutathione; PPI: Pigeon pea protein isolate; PPHPe: Pepsin hydrolyzed pigeon pea protein; PPHPa: Pancreatin hydrolyzed pigeon pea protein; PPHPp: Pepsi‐pancreatin hydrolyzed pigeon pea protein

Lipid peroxidation is of serious concern in the food industry because of its contribution to the development of undesirable off‐flavors, odors, dark colors as well as toxic products. Antioxidants function by reducing the peroxyl radical formed during lipid peroxidation to hydro‐peroxide in order to prevent the propagation of the radical chain. The ability of pigeon pea protein to inhibit lipid peroxidation was evaluated using a linoleic acid model system and the results are presented in Figure 2. The extent of peroxide formation in blank (without protein sample) increased rapidly within the first 2 days of incubation to reach its maximum absorbance (0.81) before declining. The rapid decline is supposedly due to the formation and decomposition of linoleic oxidation products such as hydroperoxides, which are unstable and gradually decomposes into secondary metabolites with the progression of incubation period. Regardless of the decline, the absorbances of the control reaction until the fifth day were still higher than values obtained in protein samples. The low inhibitory activity of reduced glutathione (GSH) has been attributed to the oxidation of GSH to glutathione sulphide (GSSG) after some days and due to the long incubation period (7 days), and its impossibility to regenerate the antioxidant form of the peptide (GSH) (Ajibola, Fashakin, Fagbemi, & Aluko, 2013). The peptides showed a good antioxidant property when compared to the control (blank) in inhibiting lipid peroxidation which is evident by the reduced absorbance or absorption intensity observed in samples with the polypeptide. PPI exhibited the weakest inhibitory activity after the second day of incubation. PPHPa was the most active in suppressing lipid peroxidation for 6 days evident by almost linear curve revealing an almost complete prevention of lipid peroxidation. This can be attributed to its high histidine content which correlates to have high lipid peroxyl radical trapping ability (Erdmann, Cheung, & Schröder, 2008). The absorbances obtained in the present study are lower than those reported for rapeseed (Mákinen, Johannson, Vegarud, Pihlava, & Pihlanto, 2012) and pumpkin (Venuste et al., 2013) hydrolysates.

### 3.4 In vitro ACE and renin inhibitory activities

**Figure 3:**
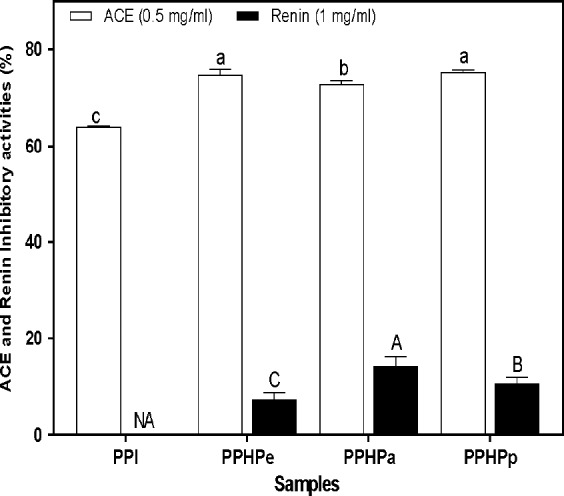
In vitro inhibition of ACE and renin activity of pigeon pea protein. Values are mean ± SD; Bars with different letters are significantly different (*p* < 0.05). PPI: Pigeon pea protein isolate; PPHPa: Pancreatin hydrolyzed pigeon pea protein; PPHPe: Pepsin hydrolyzed pigeon pea protein; PPHPp: Pepsin‐Pancreatin hydrolyzed pigeon pea protein; NA: No Activity

Pigeon pea exhibited good ACE and renin inhibitory activity (Figure 3). Elevated blood pressure is one of the major independent risk factors for cardiovascular diseases (CVD). ACE plays a crucial role in the elevation of blood pressure leading to hypertension, hence, the inhibition of ACE is important in the regulation of blood pressure. Peptides rich in hydrophobic (aromatic or branched side chains) amino acid residues are effective in inhibiting ACE activity as shown in previous studies. Superior inhibition of ACE activity was observed in PPHPp (61.82%) which may be due to higher proline content. The first two ACE inhibitory peptides (VPP and IPP) isolated from fermented milk both contained proline, which has been suggested to enhance ACE inhibition (Nakamura, Yamamoto, Sakai, & Takano, 1995). Overall, the highest ACE‐I inhibitory activity was obtained from sequential hydrolysis using pepsin and pancreatin enzymes. The results generally suggest that pigeon pea hydrolysates may have potential as antihypertensive agents. The results for PPHPp were higher than 10.53% reported for pepsin‐pancreatin lima bean hydrolysate (Ciau‐Solís, Acevedo‐Fernández, & Betancur‐Ancona, 2018). The observed differences in the ACE‐inhibitory activity have been attributed to differences in legume type, peptide mixture composition, changes in the parameters of the digestion process (enzyme, pH, time, temperature, substrate/enzyme ratio, etc.) (Barbana & Boye, 2010). In comparison with ACE inhibitory activity of other legume plants, high inhibition of the activity of key enzyme responsible for regulation of cardiovascular function was observed in the study which shows a potential benefit of using pigeon pea in the regulation of blood pressure. The present in vitro finding was further validated by an in vivo study using spontaneously hypertensive rat (SHR) model.

The activity against renin ranged from 7.35% to 14.28%. The highest degree of renin inhibitory activity was exhibited in PPHPa (14.28%). PPI exhibited no inhibitory activity against renin. The result showed a typical in vitro inhibitory difference between the blood pressure regulatory enzymes (renin and ACE), as pigeon pea peptides had more affinity for ACE than renin. This can be explained by the fact that renin assumes a more folded protein conformation, with the active sites less accessible to peptide inhibitors; hence, making it difficult to maintain active site interactions and catalytic inhibition (Yuan, Wu, & Aluko, 2007). Conversely, ACE has a more open conformation and inhibitors can easily access the active sites. Reports have demonstrated a relationship between renin and ACE, which is capable of exerting antihypertensive effect in the regulation of elevated blood pressure. Renin inhibitory activity of about 78% was reported for canola hydrolysate obtained using pancreatin (Alashi et al., 2014). Ciau‐Solís et al. (2018) reported a renin inhibitory activity of 5.5% for lima bean hydrolysate obtained using pepsin‐pancreatin sequential hydrolysis.

### 3.5 Antihypertensive effect of pigeon pea isolate and hydrolysates in spontaneously hypertensive rats (SHRs)

**Figure 4:**
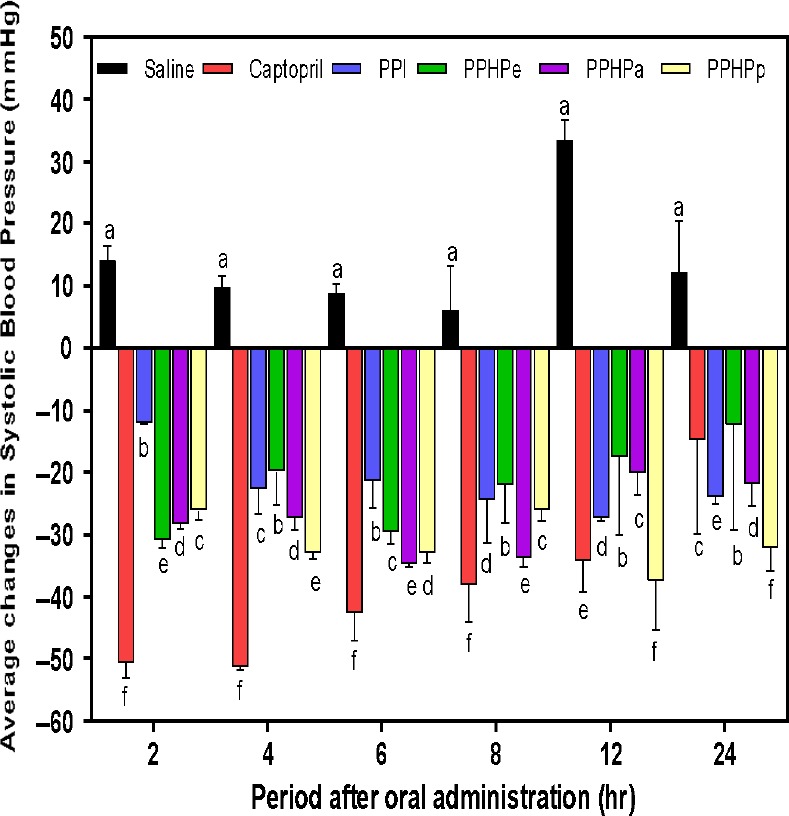
Average changes in systolic blood pressure of spontaneously hypertensive rats after oral administration of pigeon pea isolate and hydrolysates at 100 mg/kg body weight. Values are mean ± SD; Bars with different letters are significantly different (*p* < 0.05). PPI: Pigeon pea protein isolate; PPHPa: Pancreatin hydrolyzed pigeon pea protein; PPHPe: Pepsin hydrolyzed pigeon pea protein; PPHPp: Pepsin‐Pancreatin hydrolyzed pigeon pea protein

The effect of single oral administration of pigeon pea protein isolate and hydrolysate on systolic blood pressure (SBP) is shown in Figure 4. Spontaneously hypertensive rat (SHR) strain, a rat model which develops hypertension at an early stage which advances with age was used for this study. In order to validate the efficacy of pigeon pea hydrolysates as antihypertensive agents under in vivo conditions, single oral administration of pea protein on SHRs was evaluated by monitoring the reduction in systolic blood pressure over a 24 h period. Systolic blood pressure (SBP) of SHRs from negative control group orally gavage with phosphate buffer saline (PBS) was high and remained on above baseline throughout post‐oral administration time. However, SBP of rats from the positive control group orally gavage with captopril (10 mg/kg body weight) was significantly (*p* < 0.05) lower than that of PBS rats. The SBP was below baseline and the average changes reduced with increase in post‐oral administration period. Similarly, SBP of rat gavage with pigeon pea protein (isolate or hydrolysates) at a concentration of 100 mg/kg body weight was also below baseline through post‐oral administration period. After 2 h post‐oral administration, the hydrolysates were able to effectively cause a lowering of SBP above −25 mmHg. This was relative to the lowering effect (‐50 mmHg) of captopril (a potent ACE inhibitor with recognized effect as antihypertensive agent), which suggests that the hydrolysates were instantaneous in reducing blood pressure (BP) as well as confirming that the protein had been effectively digested by the enzymes employed. However, it was observed that BP lowering effect of crude isolate (PPI) was delayed within the first 2 h post‐oral administration. This may be attributed to the fact that the protein in its intact form had not been predigested hence, required the action of intestinal enzymes by the rat before the body can assimilate and utilize the peptide. All pigeon pea protein samples evaluated exhibited significant lowering of SBP between 2 and 8 h post‐administration, PPHPe had the fastest action in reducing SBP with a maximum lowering effect of −30.91 mmHg after 2 h of oral administration. The reason for this high potency may be attributed to the fact that pepsin used to generate the hydrolysate partially simulates gastrointestinal digestion. Hence, peptides generated may be resistant to further structural breakdown after oral ingestion, thus, enhanced absorption and potency in the SHRs. PPHPa showed the highest lowering effect (−34.6 mmHg) between 6 h after oral administration while PPHPp showed significant changes in SBP at 4 h (−33.0 mmHg) as well as after 12 h (−37.5 mmHg) oral administration. Significant (*p* < 0.05) reduction in SBP (−12.5 to −32.1 mmHg) was observed in the hydrolysates after 24 h of oral administration even when the effectiveness of the potent drug (captopril) had obviously declined, causing about −14.7 mmHg reduction in SBP. PPHPp exhibited strong antihypertensive effect, given the instantaneous SBP lowering effect (−26.17 mmHg) demonstrated within the first 2 h after oral administration and significantly higher lowering effect (−32.12 mmHg) exhibited after 24 h. Thus, PPHPp sustained a 24 h low SBP better than the other hydrolysates which suggests it possessed better resistance to enzymatic clearance from the blood circulatory system compared to others especially PPHPe which had the best instantaneous effect. Li, Shi, Liu, and Le (2006) reported similar reduction in SBP (−30.8 mmHg) after 6 h of single oral administration to SHRs (600 mg/kg body weight) for mung bean hydrolysate prepared with alcalase. Girgih et al. (2014) reported similar blood pressure lowering effect for a pentapeptide (IPAGV) from hemp seed 4 h after oral administration. Similar change (‐36 mmHg) in SBP was reported for another pentapeptide (LTFPG) from yellow field pea obtained 2 h after oral administration (Aluko et al., 2015). The significant decrease in blood pressure lowering activities in spontaneously hypertensive rats (SHRs) after a single oral administration demonstrates the potential of pigeon pea hydrolysates as suitable functional foods ingredient and nutraceuticals for controlling blood pressure elevation.

## 4 CONCLUSIONS

Oxidative stress constitutes an important pathophysiology in many chronic diseases, and can be controlled to reduce disease progression. The free radical scavenging, lipid peroxidation inhibitory, and ferric reducing activities of pigeon pea protein hydrolysates suggest they can attenuate tissue oxidative stress. Also, as inhibitors of ACE and renin, they can be further investigated as natural agents for managing hypertension, which is a strong risk factor for stroke. The multifunctional capacity of the pigeon pea peptides provides an opportunity to formulate functional foods and nutraceuticals with potential role in the prevention and treatment of chronic diseases.

## CONFLICT OF INTEREST

The authors declare that they do not have any conflict of interest.

## ETHICAL REVIEW

This study was approved by the institutional review board of University of Manitoba, Winnipeg, Canada. Animal ethics protocol no F16‐008/1.
